# Supplementary material for: Mechanisms governing the pioneering and redistribution capabilities of the non-classical pioneer PU.1
Source: Nat Commun. 2020 Jan 21;11:402. doi: 10.1038/s41467-019-13960-2 (PMC6972792; doi:10.1038/s41467-019-13960-2)
Supplement: Supplementary file 7 — Source data [file 41467_2019_13960_MOESM7_ESM.zip › Source_Data/Figure6/Figure6B_MotifScanOutput/ETS-specific/homerResults/motif6.similar.html]

motif6

## Information for motif6

T
A
C
G
C
A
T
G
T
C
G
A
A
C
G
T
A
C
T
G
C
G
T
A
A
T
C
G
G
C
A
T
G
T
A
C
C
G
T
A
A
C
G
T
  
Reverse Opposite:  

T
G
C
A
A
C
G
T
A
C
T
G
C
G
T
A
T
A
G
C
C
G
A
T
T
G
A
C
G
T
C
A
A
G
C
T
G
T
A
C
A
T
G
C
  

|  |  |
| --- | --- |
| p-value: | 1e-175 |
| log p-value: | -4.042e+02 |
| Information Content per bp: | 1.601 |
| Number of Target Sequences with motif | 721.0 |
| Percentage of Target Sequences with motif | 9.42% |
| Number of Background Sequences with motif | 862.1 |
| Percentage of Background Sequences with motif | 2.14% |
| Average Position of motif in Targets | 100.5 +/- 52.4bp |
| Average Position of motif in Background | 97.7 +/- 58.2bp |
| Strand Bias (log2 ratio + to - strand density) | 0.0 |
| Multiplicity (# of sites on avg that occur together) | 1.05 |
| Motif File: | file (matrix) reverse opposite |

### Similar de novo motifs found

|  |  |  |  |  |  |  |  |
| --- | --- | --- | --- | --- | --- | --- | --- |
| Rank | Match Score | Redundant Motif | P-value | log P-value | % of Targets | % of Background | Motif file |
| 1 | 0.984 | C T A G T C G A G A C T C A T G G T C A T A G C C A G T T G A C C T G A A G C T | 1e-174 | -402.410897 | 8.75% | 1.84% | motif file (matrix) |
| 2 | 0.948 | T C A G A C G T A C T G C G T A A T G C A C G T A G T C C G T A A G C T G A T C G T A C A G T C | 1e-162 | -375.068277 | 8.75% | 1.98% | motif file (matrix) |
| 3 | 0.959 | A G C T A C T G G C T A A T C G A C G T G T A C C G T A A G C T | 1e-161 | -372.016550 | 12.92% | 4.22% | motif file (matrix) |
| 4 | 0.959 | T A C G A C G T A C T G C G T A T A G C A C G T A G T C C G T A A C T G G T C A T G A C C G T A T A C G T A C G | 1e-159 | -368.192084 | 8.66% | 1.98% | motif file (matrix) |
| 5 | 0.954 | T A C G A C G T A C T G C G T A A T C G A C G T A G T C C G T A A G T C A G T C A G T C G A C T G A T C | 1e-159 | -366.765727 | 8.18% | 1.76% | motif file (matrix) |
| 6 | 0.945 | A C G T A C T G C G T A A T C G A C G T A G T C C G T A A G C T A G T C | 1e-152 | -350.777566 | 8.83% | 2.16% | motif file (matrix) |
| 7 | 0.913 | A C G T A C T G C G T A A C T G A C G T A G T C C G T A | 1e-148 | -341.338787 | 9.37% | 2.49% | motif file (matrix) |
